# Emergent Total Endovascular Repair Using Retrograde in Situ Branched Stent Grafting for Ruptured Retrograde Aortic Dissection

**DOI:** 10.5761/atcs.cr.26-00150

**Published:** 2026-09-29

**Authors:** Kenjiro Kaneko, Takao Ohki, Eisaku Ito

**Affiliations:** 1 Division of Vascular Surgery, Department of Surgery, Shinyurigaoka General Hospital, Kawasaki, Kanagawa, Japan; 2 Division of Vascular Surgery, Department of Surgery, The Jikei University School of Medicine, Tokyo, Japan

**Keywords:** retrograde aortic dissection, aortic rupture, in situ fenestration, retrograde in situ branched stent grafting, stroke prevention

## Abstract

An 85-year-old bedridden woman (modified Rankin Scale 5) presented with a contained rupture of an acute retrograde aortic dissection (Type B0,7). Because open surgery was prohibitive due to severe frailty, an emergent total endovascular repair was performed using single-branch, retrograde in situ branched stent grafting (RIBS). Two conformable GORE TAG devices established a Zone 1 seal, and the left common carotid artery was preserved by in situ needle fenestration using a percutaneous transhepatic gallbladder drainage needle and bridged with a VIABAHN VBX stent. To limit embolic risk, cerebral blood flow was transiently interrupted by manual right carotid compression, direct left carotid clamping, and left subclavian balloon occlusion. Operative time was 114 minutes, and the postoperative course was uneventful without neurological deficits. One-year computed tomography showed favorable aortic remodeling and no endoleak. Single-branch RIBS may offer an immediate, off-the-shelf salvage option for high-risk patients when open repair is not feasible.

## Introduction

Preservation of cervical branch perfusion remains one of the principal technical difficulties encountered during thoracic endovascular aortic repair (TEVAR) for aortic-arch lesions. Conventional open surgical repair for acute aortic dissection involving the aortic arch is highly invasive and carries high morbidity and mortality, particularly in frail, elderly patients with significant comorbidities.^1–3)^ Custom-made fenestrated and branched devices, although anatomically ideal, generally require a lead time of several weeks for custom fabrication, and this delay renders them impractical for truly emergent presentations. Against this background, in situ fenestration (ISF) of commercially available stent grafts emerged as an immediately deployable alternative.^4)^

We herein report a case of an elderly, high-risk patient with a contained rupture of an acute retrograde aortic dissection, diagnosed as Type B0,7 under the Society for Vascular Surgery/Society of Thoracic Surgeons (SVS/STS) classification,^5)^ in whom emergency retrograde in situ branched stent grafting (RIBS)^6)^ was performed using a percutaneous transhepatic gallbladder drainage (PTGBD) needle.

## Case Report

An 85-year-old bedridden woman with a modified Rankin Scale (mRS) score of 5^7)^ and a history of hypertension, dementia, and prior lumbar compression fractures was brought to a local hospital for sudden severe back pain. After acute aortic dissection was diagnosed on initial computed tomography (CT), she was transferred to our institution for endovascular management because open surgical repair was considered prohibitive given her frailty and comorbid burden. On arrival, her Glasgow Coma Scale (GCS) score was 14 (E4V4M6),^8,9)^ with a confused verbal response. Given her documented baseline dementia, this level of consciousness was interpreted as an acute deterioration from her chronic state. Bilateral upper-extremity blood pressures were symmetric at 212/112 mmHg, body temperature was 36.2°C, and peripheral oxygen saturation was 96% on room air.

CT angiography revealed main entry tears in the distal aortic arch and proximal descending aorta (**Fig. 1A**–**1C**, arrows). On sagittal reformats, the dissection extended retrogradely into the ascending aorta and antegradely to the level of the superior mesenteric artery, and the false lumen was partially thrombosed (**Fig. 1D**). The lesion was classified as SVS/STS Type B0,7.^5)^ The false lumen was patent along the distal arch and proximal descending aorta, whereas both the proximal segment (ascending aorta through the mid-arch) and the distal segment (mid-descending through the abdominal aorta) were completely thrombosed. All cervical and visceral branches arose from the true lumen, and there was no clinical or imaging evidence of malperfusion. The maximum diameter of the arch aneurysm was 33 mm. High-attenuation collections consistent with hematomas were observed in the pericardial and mediastinal spaces (**Figs. 1E**, **1F**, arrows). Combined with a hemoglobin drop from 11.8 to 8.5 g/dL, these findings supported the diagnosis of contained aortic rupture. Although the patient remained normotensive without overt shock at presentation, same-day emergency repair was indicated given the anatomical instability.

**Fig. 1:**
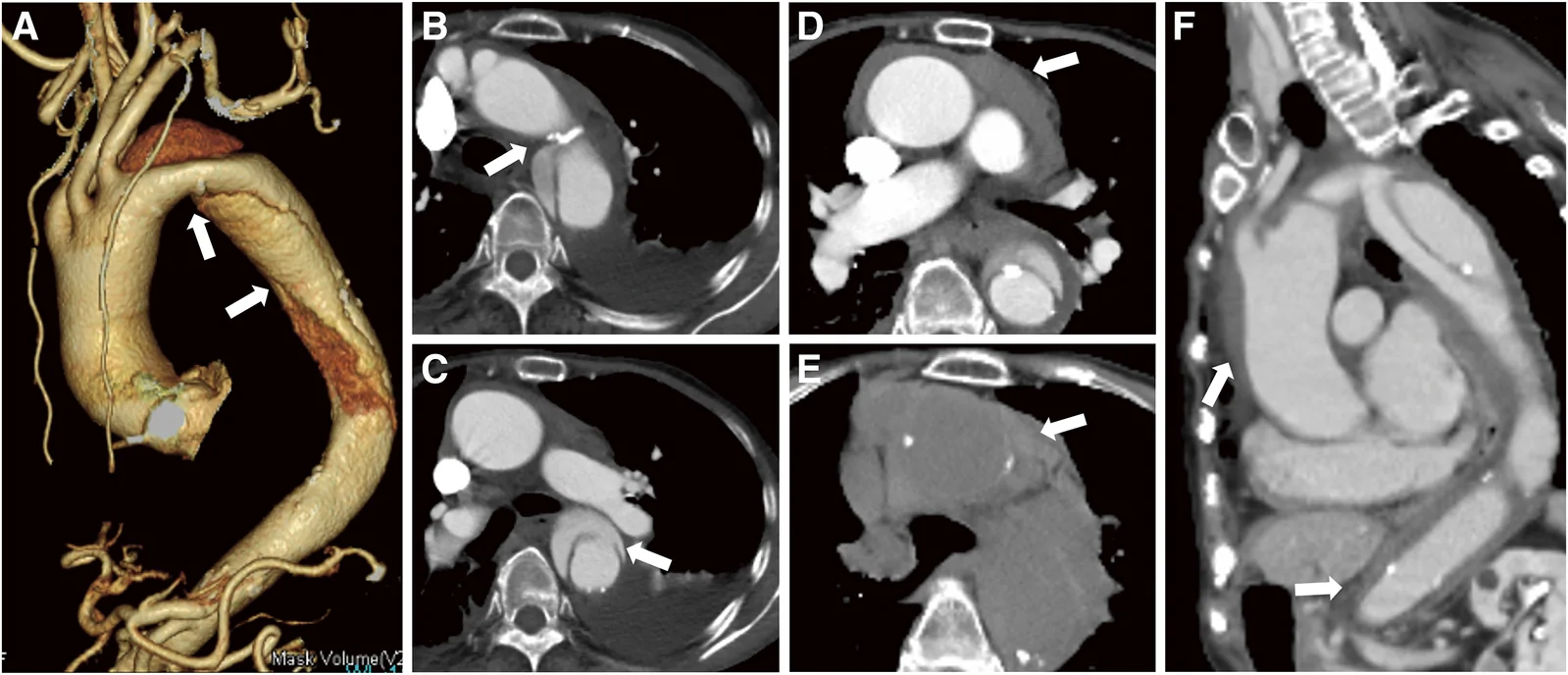
Preoperative computed tomography angiography. (**A**) Three-dimensional reconstruction image showing a patent false lumen from the aortic arch to the descending aorta, with a maximum arch aneurysm diameter of 33 mm. Arrows indicate the primary entry tears in the distal arch and descending aorta. (**B**, **C**) Axial views showing the primary entry tears in the distal arch (**B**) and descending aorta (**C**). (**D**, **E**) Axial views demonstrating high-attenuation hematoma in the pericardial space (**D**) and mediastinum (**E**). (**F**) Sagittal view showing the dissection extending from the ascending aorta proximally to the superior mesenteric artery level distally. Overall, the findings indicate a partially thrombosed retrograde aortic dissection (SVS/STS classification: Type B0,7). SVS/STS, Society for Vascular Surgery/Society of Thoracic Surgeons

Complete aneurysm exclusion required a proximal landing zone in Ishimaru Zone 1,^10)^ and therefore intentional coverage of the left common carotid artery (LCCA) origin was unavoidable. Therefore, single-branch LCCA revascularization using the RIBS technique was selected.

All procedures were performed under general anesthesia. Diagnostic aortography was initially performed via the right common femoral artery (**Fig. 2A**). The main endograft was delivered through the left common femoral artery, and an occlusion balloon was positioned within the left subclavian artery (LSA). A Conformable GORE TAG Thoracic Endoprosthesis (CTAG; 31 × 150 mm; W. L. Gore & Associates, Flagstaff, AZ, USA) was deployed in the descending thoracic aorta, and a second CTAG device (40 × 200 mm) was subsequently deployed in Zone 1 as the proximal landing zone.

**Fig. 2:**
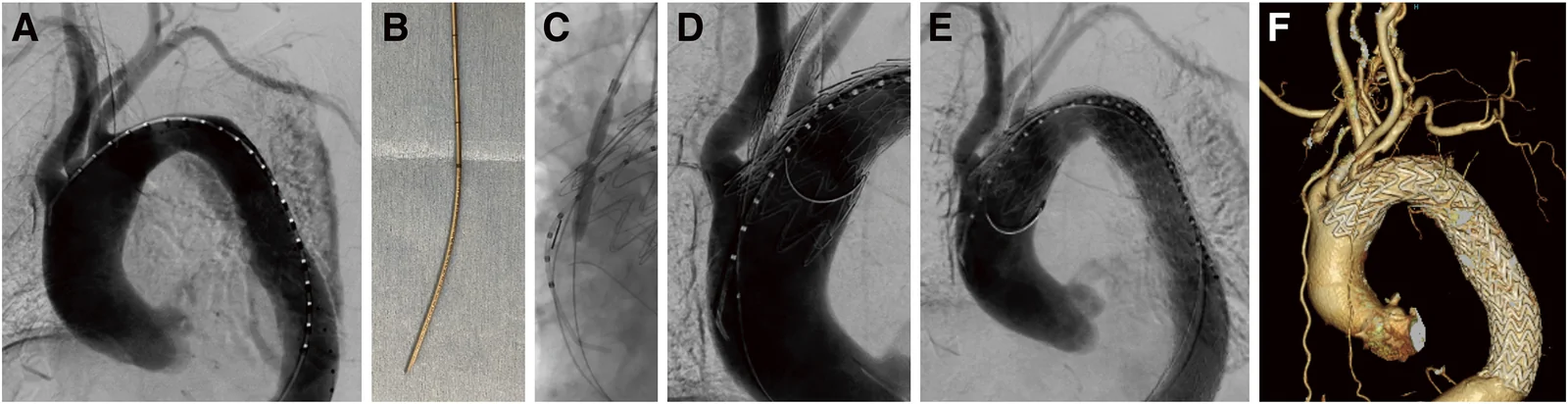
Intraoperative angiography and postoperative three-dimensional computed tomography. (**A**) Initial intraoperative angiogram. (**B**) The Hanako PTGBD needle (30 cm; Koken Co., Ltd.) used for the puncture. The tip is manually bent to an optimal angle tailored to the anatomical angle between the target carotid artery and the aorta. (**C**) Dilation of the fenestration using an Athletis PTA balloon dilatation catheter (7 × 40 mm; Boston Scientific). The presence of a “waist” during the early phase of balloon inflation confirms that the wire accurately penetrates the stent-graft fabric. (**D**) Deployment and dilation of the GORE VIABAHN VBX Balloon-Expandable Endoprosthesis (8 × L-59 mm; W.L. Gore & Associates). The proximal end of the VBX protrudes sufficiently (approximately 2 cm) into the aortic lumen. (**E**) Completion angiogram. (**F**) Three-dimensional computed tomography image one year after RIBS showing no endoleak and favorable aortic remodeling with complete thrombosis of the false lumen. PTGBD, percutaneous transhepatic gallbladder drainage; PTA, percutaneous transluminal angioplasty; RIBS, retrograde in situ branched stent grafting; VBX, VIABAHN VBX

After surgical exposure of the LCCA, an 8-Fr BRITE TIP sheath (23 cm; Cordis Corporation, Miami Lakes, FL, USA) was retrogradely advanced through the vessel. A PTGBD needle (Hanako, 30 cm; Koken Co., Ltd., Tokyo, Japan) was shaped ex vivo by manual bending to match the take-off angle of the LCCA (**Fig. 2B**) and advanced over a stiff Radifocus Guide Wire M (Terumo Corporation, Tokyo, Japan) to the carotid bifurcation. During half deployment of the second CTAG, the sheath tip was advanced against the CTAG fabric to confirm the contact angle. Once the graft was fully expanded, the PTGBD needle was advanced through the fabric to create a pathway for the delivery of the stiff wire into the ascending aorta. The needle was then removed, and the inner dilator of a 7-Fr Radifocus Introducer II (25 cm; Terumo Corporation) was passed across the fenestration.

The fenestration was serially dilated using an Athletis PTA balloon dilatation catheter (7 × 40 mm; Boston Scientific, Marlborough, MA, USA). A residual “waist” on initial low-pressure inflation confirmed the engagement of the balloon with the punctured fabric (**Fig. 2C**). Serial inflation to an ultra-high pressure of 30 atm was followed by control aortography, which showed brisk antegrade flow into the LCCA. After balloon dilatation of the CTAG, a GORE VIABAHN VBX Balloon-Expandable Endoprosthesis (8 × L-59 mm; W. L. Gore & Associates) was deployed across the fenestration to bridge the LCCA and aortic lumen (**Fig. 2D**). Completion angiography confirmed patent antegrade LCCA flow with no residual false lumen perfusion (**Fig. 2E**).

The operative parameters were as follows: procedure time, 114 min; estimated blood loss, 50 mL; fluoroscopy time, 22 min; and iodinated contrast volume, 273 mL. The patient was extubated on the day of surgery and discharged from the intensive care unit on postoperative day 2. No clinical or radiographic evidence of cerebral infarction was observed, and no other new neurological deficits developed during hospitalization. Oral intake was uneventfully reintroduced, and the patient was transferred to another facility for rehabilitation on postoperative day 22. Follow-up CT at 1 year showed favorable aortic remodeling with complete false lumen thrombosis, and there was no evidence of endoleak, branch stent occlusion, or structural graft failure (**Fig. 2F**).

## Discussion

The present case involves a contained rupture of an acute aortic dissection in an elderly, bedridden patient (mRS 5).^7)^ According to the 2020 SVS/STS reporting standards,^5)^ this lesion is classified as a Type B0,7 dissection because the main entries were located in the distal arch and descending aorta, and the thrombosed dissection extended retrogradely into the ascending aorta and antegradely to the level of the superior mesenteric artery. In this patient, the development of a contained rupture might be related to the partial thrombosis of the false lumen observed on the initial CT.^11)^ As Tsai et al. noted, partial thrombosis can elevate false lumen pressure and is considered a risk factor for aortic expansion and mortality.^12)^ Given the prohibitive risk of open surgery for this patient, endovascular repair using the RIBS technique was chosen as a less invasive alternative to exclude the main entries without the physiological burden of extracorporeal circulation, and the procedure was successfully completed in 114 min.

Preoperative determination of the landing zone and intraoperative prevention of cerebral infarction are key considerations in performing this procedure. Preoperative imaging revealed a 14-mm segment between the proximal extent of the dissecting arch aneurysm and the origin of the LCCA. Because this length was insufficient for a Zone 2 landing, a Zone 1 landing requiring single-branch LCCA reconstruction was chosen. As previously reported, the presence of a mural thrombus in the aortic arch is a significant risk factor for cerebral infarction during TEVAR, making a Zone 0 landing preferable in such scenarios to avoid embolization.^13)^ However, because a mural thrombus was absent in this patient and the contained rupture necessitated a simpler and more rapid procedure, a Zone 1 approach was chosen. Furthermore, to prevent cerebral infarction during insertion of the main stent graft, temporary blood flow interruption was performed. In cases of single-branch RIBS, balloon occlusion at the origin of the LSA, direct clamping of the surgically exposed LCCA, and manual compression of the right common carotid artery were performed (**Fig. 3**). Although completely preventing microembolization is technically challenging, this blood flow interruption technique is routinely performed in all arch endovascular interventions at our institution to suppress cerebral inflow, thereby minimizing the entry of potential emboli into the carotid arteries during main stent-graft passage.^14)^ Additionally, cerebral angiography is performed before and after the RIBS procedure to check for the presence of major embolic occlusion. If a major embolus is detected, rescue procedures, such as catheter-directed mechanical thrombectomy, can be immediately performed in our hybrid operating suite. In the present case, completion angiography showed no major embolic events, and the postoperative course was uneventful.

**Fig. 3:**
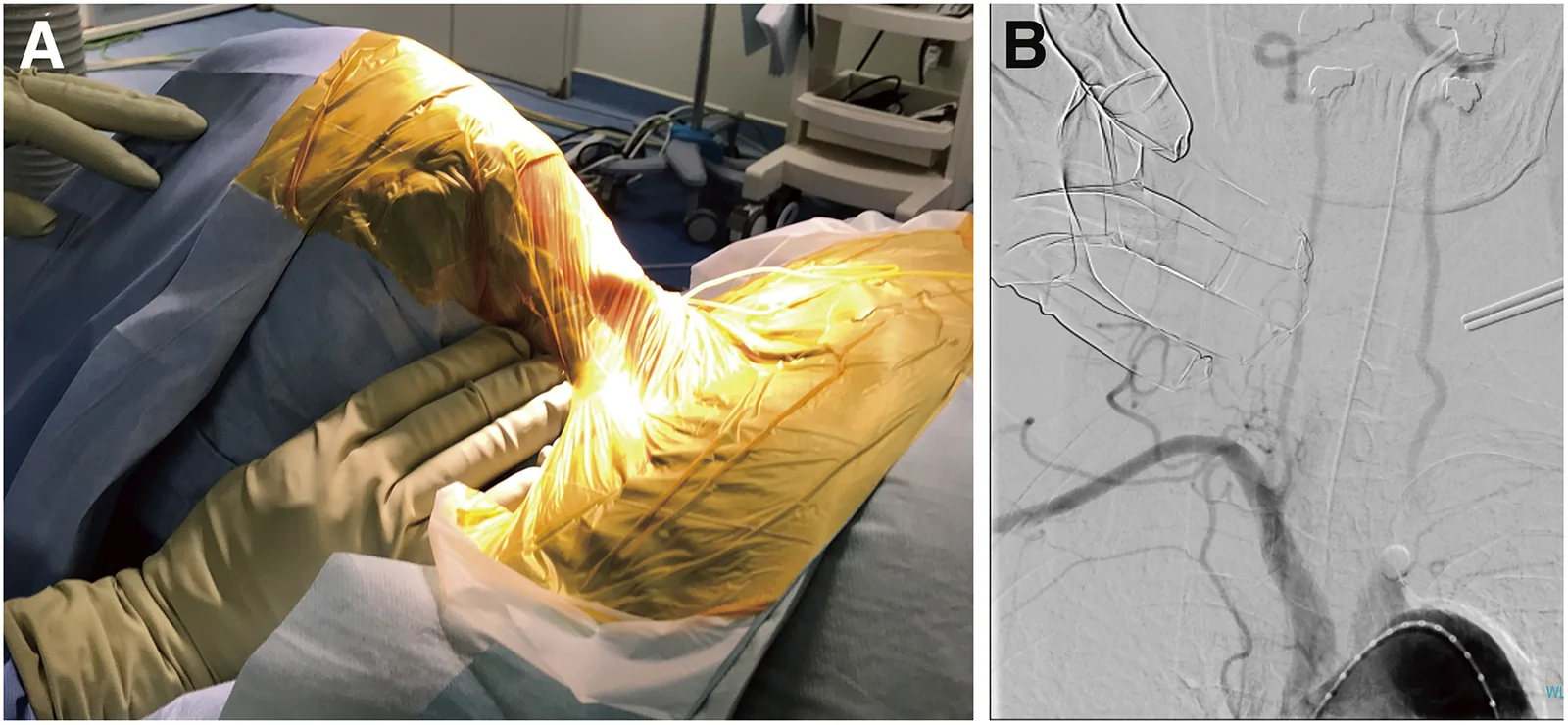
Blood flow interruption techniques for stroke prevention. (**A**) Manual compression of the right common carotid artery. This maneuver demonstrates temporary blood flow interruption to prevent cerebral embolism during the passage of the stent graft through the aortic arch. (**B**) Angiogram during blood flow interruption in the present case (single-branch RIBS). Blood flow is interrupted by manual compression of the right common carotid artery, direct clamping of the left common carotid artery, and balloon occlusion of the left subclavian artery. At this time, only cerebral perfusion via the vertebral arteries is maintained, showing retrograde contrast flow in the left vertebral artery (subclavian steal). RIBS, retrograde in situ branched stent grafting

The selection of graft materials and devices is important for procedural reliability and long-term durability of the ISF technique. *In vitro* studies suggest that expanded polytetrafluoroethylene (ePTFE) materials maintain their dimensions after fenestration with less alteration compared to polyethylene terephthalate (PET) fabrics.^15,16)^ In this case, an ePTFE-based CTAG device was used, which has been reported to show minimal fabric fraying after puncture.^6)^ During the fenestration process, penetrating the graft fabric as perpendicularly as possible is important to ensure reliable graft puncture. For this purpose, the PTGBD needle needs to be manually bent *ex vivo* to match the patient’s anatomy (**Fig. 2B**). Furthermore, the silicone-coated BRITE TIP sheath placed in the carotid artery is characterized by its slip-resistant nature. This feature helps to prevent unexpected sheath dislodgement during the procedure. Additionally, its high rigidity facilitates carotid artery straightening, thereby improving the ease of puncture. For subsequent enlargement of the fenestration, previous studies have cautioned that cutting balloons may exacerbate fabric tearing, potentially leading to late type III endoleaks.^6,15,17)^ Therefore, a stepwise dilation strategy using a sheath inner dilator followed by a high-pressure balloon was adopted to minimize graft injury and provide an adequate seal at the junction with a balloon-expandable covered stent (VBX).

Our institutional strategy for the endovascular management of aortic arch pathologies includes hybrid approaches, such as debranching TEVAR, carotid–carotid bypass, and the chimney technique. To address complications associated with these modalities, including cerebral infarction and gutter leaks, our institution adopted the RIBS procedure.^18)^ As mentioned, manufacturing delays limit the utility of custom-made devices in emergencies.^4)^ Therefore, the RIBS technique serves as a practical, immediately deployable alternative.

However, RIBS is not universally applicable. Anatomical features that weigh against the technique include mural thrombus in the intended proximal landing zone, an aortic diameter greater than approximately 42 mm at that level, and a near-parallel takeoff of the target cervical branch relative to the aortic centerline, which makes perpendicular needle-puncture technically difficult.^6)^ Several limitations of this study should be acknowledged. First, this was a single case with only 12 months of follow-up, and the findings cannot be generalized. Second, the long-term behavior of an unreinforced ISF remains incompletely characterized, and late endoleak, in-stent stenosis, and branch stent occlusion cannot be excluded. Further clinical experience and lifelong imaging surveillance are needed before the safety and durability of single-branch RIBS for ruptured lesions can be established.

## Conclusion

For elderly patients at high surgical risk, single-branch LCCA reconstruction using the RIBS technique with off-the-shelf devices and a PTGBD needle was feasible and provided timely aneurysm exclusion when open repair and custom branched devices were not options. Careful preoperative planning and a considered choice of graft material and a bridging stent may have contributed to the technical success and favorable short-term aortic remodeling in this case. Whether this experience can be reproduced in larger studies requires further investigation.

## Declarations

### Use of artificial intelligence (AI) tools

Generative AI (NotebookLM) was used for language translation to improve the readability of this manuscript. The authors reviewed the content and take full responsibility for the final manuscript.

### Data availability

Data sharing is not applicable to this article.
